# Using MALDI-TOF spectra in epidemiological surveillance for the detection of bacterial subgroups with a possible epidemic potential

**DOI:** 10.1186/s12879-021-06803-3

**Published:** 2021-10-28

**Authors:** Audrey Giraud-Gatineau, Gaetan Texier, Pierre-Edouard Fournier, Didier Raoult, Hervé Chaudet

**Affiliations:** 1 Institut Hospitalo-Universitaire Méditerranée-Infection, 19-21 Boulevard Jean Moulin, 13005 Marseille, France; 2 Aix Marseille Univ., IRD, AP-HM, SSA, VITROME, IHU Méditerranée Infection, Marseille, France; 3 Aix Marseille Univ., IRD, AP-HM, MEPHI, Marseille, France; 4 Centre d’Epidémiologie et de Santé Publique des Armées (CESPA), Marseille, France; 5 Assistance Publique Hôpitaux de Marseille, Marseille, France

**Keywords:** MALDI-TOF, Epidemiological surveillance, Cluster analysis, Epidemic

## Abstract

**Background:**

For the purpose of epidemiological surveillance, the Hospital University Institute Méditerranée infection has implemented since 2013 a system named MIDaS, based on the systematic collection of routine activity materials, including MALDI-TOF spectra, and results. The objective of this paper is to present the pipeline we use for processing MALDI-TOF spectra during epidemiological surveillance in order to disclose proteinic cues that may suggest the existence of epidemic processes in complement of incidence surveillance. It is illustrated by the analysis of an alarm observed for *Streptococcus pneumoniae.*

**Methods:**

The MALDI-TOF spectra analysis process looks for the existence of clusters of spectra characterized by a double time and proteinic close proximity. This process relies on several specific methods aiming at contrasting and clustering the spectra, presenting graphically the results for an easy epidemiological interpretation, and for determining the discriminating spectra peaks with their possible identification using reference databases.

**Results:**

The use of this pipeline in the case of an alarm issued for *Streptococcus pneumoniae* has made it possible to reveal a cluster of spectra with close proteinic and temporal distances, characterized by the presence of three discriminant peaks (5228.8, 5917.8, and 8974.3 m/z) and the absence of peak 4996.9 m/z. A further investigation on UniProt KB showed that peak 5228.8 is possibly an OxaA protein and that the absent peak may be a transposase.

**Conclusion:**

This example shows this pipeline may support a quasi-real time identification and characterization of clusters that provide essential information on a potentially epidemic situation. It brings valuable information for epidemiological sensemaking and for deciding on the continuation of the epidemiological investigation, in particular the involving of additional costly resources to confirm or invalidate the alarm.

***Clinical trials registration*:**

NCT03626987.

**Supplementary Information:**

The online version contains supplementary material available at 10.1186/s12879-021-06803-3.

## Background

Epidemiological surveillance systems have a central role in order to control and manage infectious diseases [1, 2]. Since 2013, the Hospital University Institute Méditerranée infection (IHU-MI) has implemented an epidemiological surveillance system named MIDaS (for Mediterranée Infection Data Warehousing and Surveillance) made of five syndromic surveillance sub-systems. This system is based on the systematic recording of routine results issued from clinical microbiology and virology laboratories, which are not specifically done for surveillance purpose [3], including identification at species level and possibly phenotypic or genomic characters. Data from other information systems are also collected, such as spectra files generated by the Matrix Assisted Laser Desorption Ionization–Time of Flight (MALDI-TOF) mass spectrometers used for bacterial and fungal species routine identification [4]. MIDaS automatically and systematically analyses the number of bacteria identifications in search of abnormal increases, corresponding to “surveillance alarms”. Each week during a staff an evaluation of these alarms is done in order to decide how to deal with them, doing a epidemiologic sensemaking that we have previously conceptualized under the term “situation diagnosis” [5]. During this situation diagnosis, MIDaS also helps to contextualize the alarm, allowing an “in silico” investigation based on sample and patient characteristics.

Recent publications have demonstrated that species-level surveillance alone is often insufficient to carry out the situation diagnosis [6–9] because a same bacterial species may present a great diversity of subspecies with strong variations in clinical and epidemiological expression, each of them possibly being an epidemics [10]. The search for specific genetic markers or the use of antibiograms make it possible to detect this kind of subspecies outbreaks, but requires sometime extensive extra works.

Nowadays, MALDI-TOF MS is used in routine bacterial identification and for retrospective epidemiological investigations [10–14]. It appears to be an answer to these more time-consuming and tedious laboratory techniques. Retrospective studies based on spectra clustering revealed the proteinic similarity of strains sharing the same geographical area and the same epidemic features [11–14] or the dissimilarity between epidemic strain and usual species spectra [10]. However, to our knowledge, no study reports the use of MALDI-TOF MS during a routine epidemiological surveillance activity [5, 15].

The objective of this paper is to supplement the epidemiological surveillance system already in function with a MALDI-TOF spectra analysis at a sub-population level, and allowing an in silico epidemiological pre-investigation. For this purpose, we will present a pipeline for processing MALDI-TOF spectra during epidemiological surveillance in order to disclose a latent clustering of a species, which may suggest the existence of epidemic processes. This description will be illustrated by the analysis of an alarm observed for *Streptococcus pneumoniae*.

## Materials and methods: description of the pipeline

**Fig. 1:**
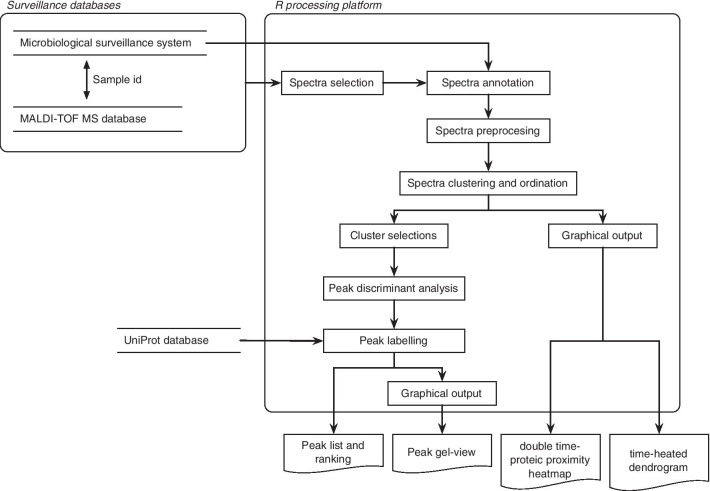
Process flow of the matrix-assisted laser desorption ionization mass spectrometry mass spectra analysis, from databases to system outputs

The spectra-based surveillance system relies upon a microbiological surveillance system associated with a MALDI-TOF MS database. The overall process flow is described in Fig. 1.

### Microbiological surveillance system: BALYSES subsystem

The whole activity of bacterial clinical microbiology of the IHU-MI, covering the 4 public and university hospitals of the Assistance Publique – Hôpitaux de Marseille (AP-HM) in Marseille, France, is monitored since February 2014 by an automated surveillance system named BALYSES [7] (Bacterial real-time Laboratory-based Surveillance System), which is one the five MIDaS (for Mediterranée Infection Data Warehousing and Surveillance) subsystems. Connected to the laboratory information system, this surveillance system is based on a dedicated data warehouse gathering microbiological analysis results (sample id, requesting department, date, sampling, analysis, result, possible antibiotic susceptibility testing, possible antibiotic resistance phenotype, bacterial co-identifications) and patient-related information (anonymized patient id, age, sex, home postal code, anonymized hospital stay id, department stay date, death during hospitalization). It allows a systematic weekly detection of outbreaks for all bacterial species included in the database using CUSUM algorithms [16], the monitoring of trends for the sampling activity of the 15 most frequent bacterial species, and tracking of rare or new bacterial species. We have calculated the optimal parameters for our surveillance system by applying the method proposed by Rolfhamre [17]. The parameters of CUSUM are k = 0.5 and h = 1.5 with a reference history of 6 months. The use of the CUSUM aberration detection method at the species level is used as a guide to visualize a potential homogeneous increase of a bacterial subpopulation by means of clustering of MALDI-TOF spectra.

### MALDI-TOF MS database from IHU-MI

Since 2014, February 1st, the MALDI-TOF database has gathered around 900,000 spectra performed at the IHU-MI for the routine bacterial identifications of the AP-HM.

After culturing on blood or chocolate agar (depending on the species and stopped in the middle of log phase), a single colony is directly applied in on 2 or 4 spots on ground steel targets, air dried, overlaid with α-cyano-4-hydroxycinnamic acid matrix solution in 50% of acetonitrile and 2.5% of trifluoroacetic acid and air dried following the agreed protocol. All bacterial spectra are acquired using 3 Bruker Daltonics Microflex MALDI-TOF MS with FlexControl Software, using the default settings (positive linear mode within the m/z range of 2 to 20 kDa, laser frequency 60 Hz; ion source 1 voltage, 20 kV; ion source 2 voltage, 16.7 kV; lens voltage, 7.0 kV), and 240 laser shots at 60 Hz. Culture standardization is required for allowing spectra comparability within a same species. Bruker BioTyper® software allows the comparison between the spectrum and a reference database and leads to the bacterial species routine identification when the score threshold is ≥ 2.0. The Bacterial Test Standard (BTS) which is a solution of *Escherichia coli* DH5 alpha with two additional proteins, is used as a positive control and the matrix solution as a negative control for identification. Automata calibration is regularly performed as described by Bruker’s protocol using the BTS.

All MALDI-TOF MS spectra (‘fid’ files) and their parameter files (‘acqu’ files) produced during the identification process are extracted from our laboratory automata and saved in a specific file system storage included in the MIDaS data warehouse.

### MALDI-TOF MS analysis

Our spectra processing platform is based on a homemade program written in R [18] and mainly using the following packages: MALDIquant v1.16.2 [19] for spectra reading and quantitative analysis, seriation v1.2-2 [20] for dendrogram ordering, and BinDA v1.0.3 [21] for the protein peak discriminant analysis using binary predictors.

#### Spectra selection

For investigating an alarm, the related surveillance database records and their associated spectra are selected using a suitable request (e.g. selecting the species concerned by the alarm, the time window corresponding to the alarm, some antibiotic susceptibility indicator, some home location, some hospital department…), along with a time window extension (over a maximal period of 4 months) for including a sufficient non-epidemic control samples for contrasting the spectra associated with the epidemiological alarm. This delay may be shorter if the number of spectra is too huge to make the clustering readable, as for *Escherichia coli* or *Staphylococcus aureus*. The limit usually used in this case is about 1500 spectra. Conversely, for rare bacterial species (identified less than 10 times per year), all spectra from the database can be included in the analysis. During the selection process spectra quality is taken in account: only spectra of sufficient quality in terms of saturation and noise [22] and with plate controls (BTS) required for spectra deviation correction (as described below) are included in the analysis. The samples as well as the patients are well deduplicated.

#### Spectra processing

The selected spectra are imported into the analysis platform and are then injected into a 4-step workflow, which is described below. The spectra processing includes normalization [19], double alignment of spectra [23], Main Spectrum Profiles (MSP) and intensity matrix building. During these steps, the signal to noise ratio (SNR) was 2 and was used as a peak detection threshold, the peaks with a SNR < 2 being considered as noise.

As described by Gibb and Strimmer [19], the normalization is made of intensity transformation (square root method), smoothing (moving average with half window size 12), baseline correction (Statistics-sensitive Non-linear Iterative Peak-clipping algorithm, 100 iterations) and intensity recalibration (on the maximal intensity peak).

The 8 reference peaks (3637.8, 5096.8, 5381.4, 6255.4, 7274.5, 10,300.1, 13,683.2, 16,952.3 Da) of the BTS, required for each target plate, are used for a first alignment (quadratic warping function) aiming at controlling automata-dependant drift. Spectra with reference peaks out of the built-in Microflex tolerance window (300 ppm) are dropped. Using the species typical peak composition described in our panspectrome database [23], a second alignment of the spectra based on their species-specific common peaks is then done (quadratic warping function with 0.005 tolerance).

Technical replicates are averaged into main spectrum profiles (MSP), and species specific common peaks are removed in order to increase the contrast between these spectra, which belong to the same bacterial species [23].

An intensity matrix, describing the intensity of spectra peaks for each MSP, and built as recommended by S. Gibb [21], is the final deliverable of this process.

#### Spectra clustering

The next step is the hierarchical clustering of the intensity matrix using Bray–Curtis distance and Ward agglomeration with ordination (or seriation). The ordination is based on the Gruvaeus–Wainer method [24], which orders the leaves at each merging step such the leaves at the edges of each cluster are beside the more similar ones, ensuring the unicity of the dendrogram. Time distances between dendrogram leaves are also calculated during this step.

The results of this clustering step are presented using 2 specific graphics: a time-heated dendrogram and a time-protein double proximity heatmap. Their aim is to support epidemiological inference based on the MSP closeness in terms of proteinic and temporal distances, suggesting the possible epidemiological relations between isolates, as elaborated by Sintchenko et al. [25]. In the time-heated dendrogram, each leaf label is coloured with a heat scale according to the case occurrence time. More the case is recent and more the color is “hot”, from blue to red. Isolates possibly belonging to a same epidemiological event are represented in the dendrogram by subtrees with labels showing the same colour. The time-protein double proximity heatmap combines a first half-matrix showing proteinic distances with a second half-matrix coloured in accordance with the time distance between MSP (Fig. 4, subtree A). The double heatmap is a possible alternative illustration where groups of MSP with close proteinic-temporal distances appear as hot colour squares along the matrix diagonal.

#### Spectra characterization

Characterization of MSP belonging to a group is done by contrasting this group against the other MSP with a discriminant analysis on protein peaks. For this purpose, we rely on the Gibb and Strimmer’s method for differential protein expression and prediction based on binary discriminant analysis (BinDA) [21]. This method dichotomizes the intensity vector of each peak using the maximisation of the Kullback–Leibler divergence, before finally ranking them according to their discriminating power. All top-ranked peaks are automatically checked against the UniProt database (http://www.uniprot.org/) using its representational state transfer (REST) programmatic access. A mass fluctuation of ± 2 Da is allowed for the matching. For each top-ranked peak, prediction errors for group separation are estimated using cross-validation procedures [26].

### Ethic information

This study has been allowed by the French Data Protection Authority (CNIL decision DR-2018-177), and declared on ClinicalTrials.gov Protocol Registration and Result System (id: NCT03626987).

## Results

### Surveillance system activity

At the date of February 2020 (316 weeks since 2014, February 1st), the microbiological surveillance database includes 287,679 bacterial identifications for 559 different species, from 237,196 clinical samples and 100,729 patients (137,625 hospital stays). The associated MALDI-TOF datawarehouse gathers 929,740 MALDI-TOF MS spectra. The database increases at a weekly rate of about 12,000–13,000 samples and 1000 bacterial identifications for 1000–1300 new patients. The three most represented sample are urine samples (79,528 samples, 33.5% of the total) followed by blood samples (43,188 samples, 18.2%) and respiratory samples (25,966 samples, 10.9%). The ten most identified bacterial species are *Escherichia coli* (61,734 strains, 21.5% of the total), *Staphylococcus aureus* (46,791 strains, 16.3%), *Staphylococcus epidermidis* (22,180 strains, 7.7%), *Pseudomonas aeruginosa* (19,789 strains, 6.9%), *Klebsiella pneumoniae* (18,722 strains, 6.5%), *Enterococcus faecalis* (12,723 strains, 4.4%), *Enterobacter cloacae* (10,091 strains, 3.5%), *Streptococcus agalactiae* (8124 strains, 2.8%), *Gardnerella vaginalis* (7773 strains, 2.7%) and *Proteus mirabilis* (5934 strains, 2.1%). *Streptococcus pneumoniae* is involved in 2509 strains (0.9%).

### Illustrative alarm analysis

**Fig. 2:**
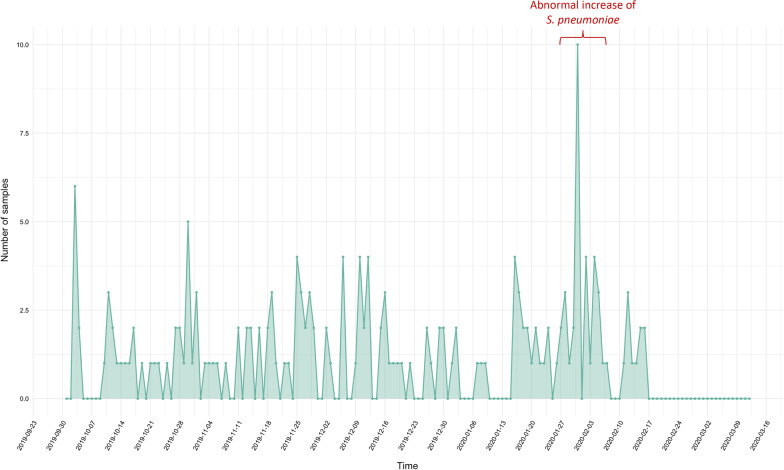
Number of *Streptococcus pneumoniae* samples from 10 October 2019 to 12 March 2020 at AP-HM, Marseille

BALYSES surveillance system found an abnormal increase of *Streptococcus pneumoniae* identifications from January 31th to February 9th 2020 (5–6th weeks), with 17 cases for 12 expected. *S. pneumoniae* is known to be amongst the worldwide leading cause of death due to infectious diseases [27] and has been implicated in 22 alarms in our system since February 2014. For the purpose of the MALDI-TOF investigation of this alarm, and following the protocol described above, we selected the spectra with a request that searched for the bacterial species concerned by the alarm (*Streptococcus pneumoniae*) along with a time window extended from October 1st, 2019 to February 16th, 2020, and without restriction on the home location, hospital department or a phenotypic character (Fig. 2). The 17 patients having caused the *S. pneumoniae* alarm were 13 men and 4 women. Their mean age was 35.6 years, and the length of their hospital stay was 6.2 days in average. *S. pneumoniae* was mostly identified in blood cultures (N = 7, 41.2%), respiratory samples (N = 6, 35.3%) and deep samples (N = 2, 11.8%).

During the extended period considered for this analysis, 213 *S. pneumoniae* identifications were performed, corresponding to 171 samples from 136 patients and 138 hospital stays. A total of 644 MALDI-TOF spectra were associated in the spectra datawarehouse. After application of quality criteria, 421 (65.4%) spectra related to 123 patients (125 hospital stays) were retained for further analysis. During the spectra processing, these spectra were grouped in 125 MSPs (Main Spectrum Profile), producing an intensity matrix of 125 rows (MSPs) and 152 columns (peaks) as final result.

**Fig. 3:**
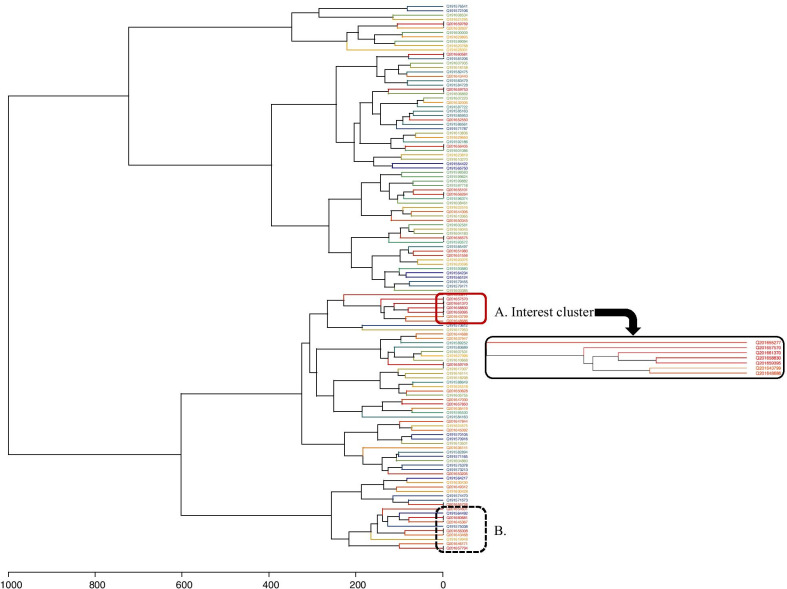
Complete Time-heated dendrogram of the 125 main spectrum profiles of *S. pneumoniae* illustrating the use of leaf label coloring. The colorscale shows the case recency, from to most ancient (blue color) to the most recent (red color), and then case concomitance. The interest cluster (red square) is indicated with an enlargement of the dendodragrm illustrating the possible leaf labelling using surveillance data. The stars near the leaves are the spectra involved in the alarm emitted by BALYSES

**Fig. 4:**
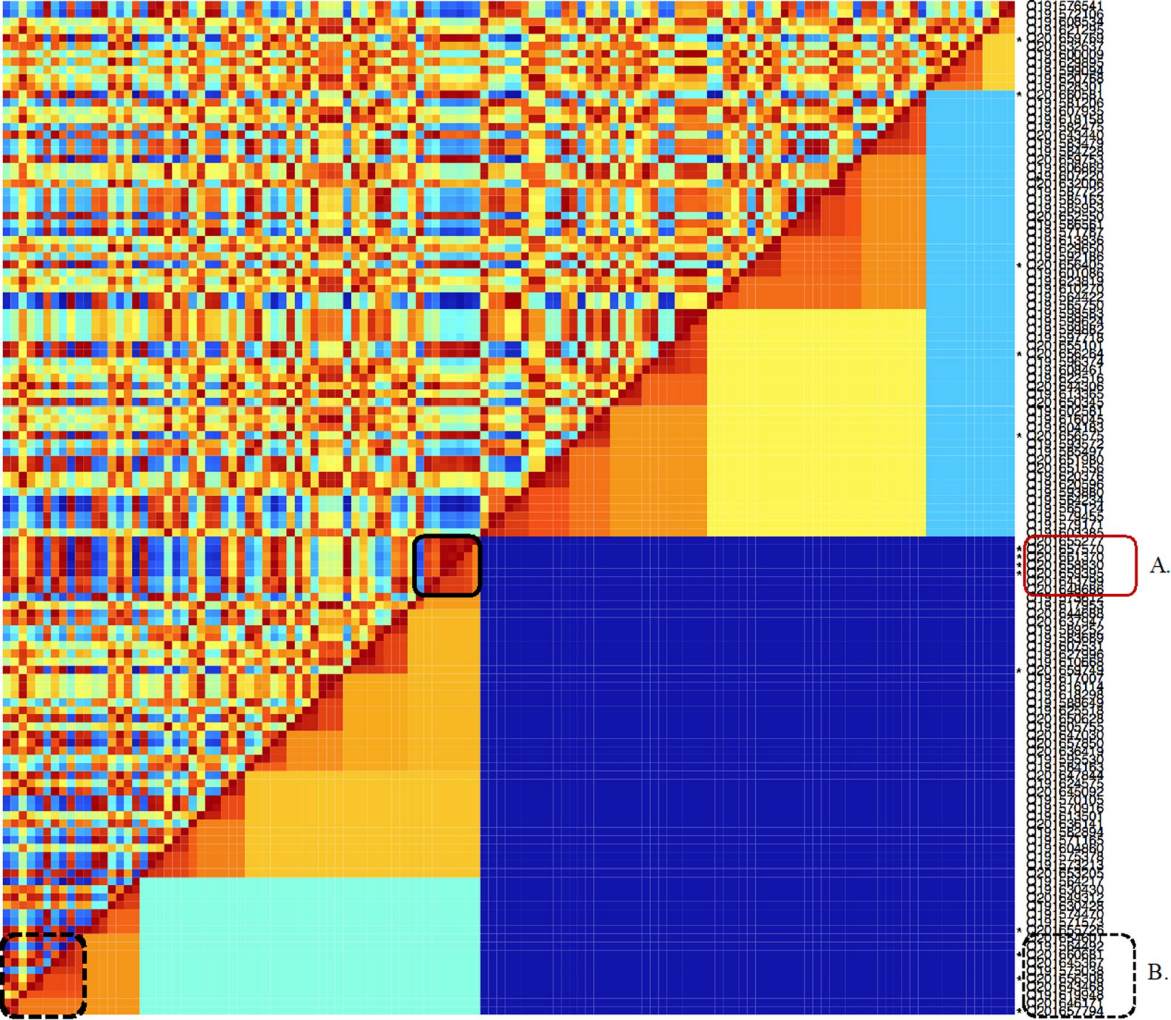
Double time-protein proximity heatmap resulting from the analysis of the 125 main spectrum profiles (MSP) of *S. pneumoniae*. The interest cluster is indicated. The bottom-right hemi-matrix shows the samples’ proteinic proximity. The top-left hemi-matrix shows the samples’ time concomitance. The colorscale is the same for the two hemi-matrices: blue corresponds to the largest distances and red to the closest ones. Spectra with closed time-protein distances appears as a square in hot colour along the matrix diagonal, as subtree A. The stars near the leaves are the spectra involved in the alarm emitted by BALYSES. Subtree D has a less epidemiological interest with a temporal heterogeneity

The results of the spectra clustering phase are presented in Figs. 3 and 4. Due to the color code used for the representations, patients involved in the surveillance alarm are included in dendrogram’s red labels. They are mainly concentrated in 2 subtrees (subtrees A and B), which may be associated to two simultaneous epidemiological events.

The subtree A gathers 7 MSPs produced during the previous 3 weeks, while the subtree B is a grouping of 10 MSPs produced over a 4 months period, and less pertinent for the alarm investigation. The related surveillance database data show that all MSPs of subtree A are coming from different patients, with 4 associated to patients involved in the alarm, and 4 MSPs coming from respiratory samples, 2 from deeper samples and 1 from skin sample. The antibiotic tests from these 7 samples are presented in Additional file 1: Table S1. In our in silico epidemiological pre-investigation, the 7 patients of subtree A were from different hospital wards, ruling out a possible nosocomial epidemic at first sight (Additional file 1: Figure S1). Nevertheless, two patients were hospitalized in the same medical unit 10 days apart. The first patient died. All the individuals came from the same region. Five of them came from the same department, while the other two came from two neighboring departments. The time-protein double proximity heatmap (Fig. 4) confirms the epidemiological interest of subtree A, showing a corresponding ‘hot’ square.

**Fig. 5:**
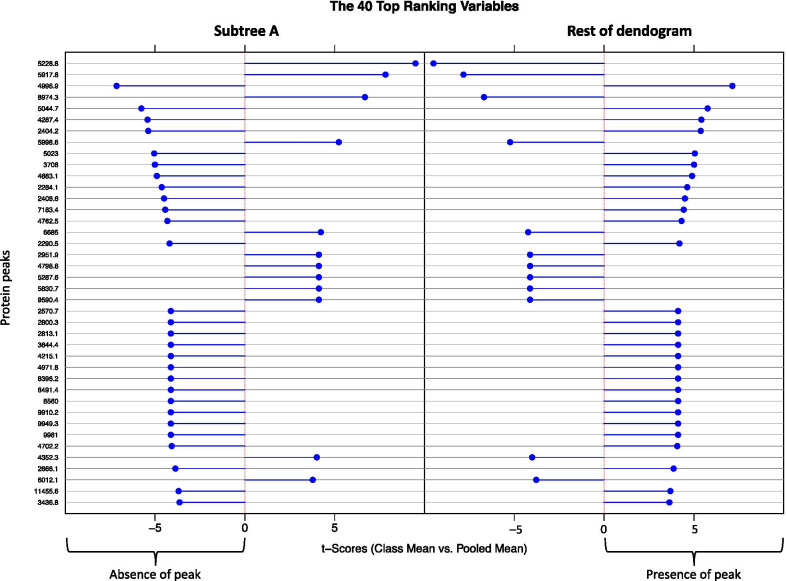
Binary discriminant analysis of the 125 main spectrum profiles (MSP) of *S. pneumoniae* showing the 40 top ranking peaks contrasting the 7 samples belonging to interest cluster against the other ones. Peaks are indicated using their m/z. For each selected peak the entropic ranking t-score is represented, positive when the peak is associated with the group

We have tried to find what peaks were able to contrast the MSPs of subtree A with the rest of the dendrogram, using a binary discriminant analysis (Fig. 5). In this representation, a positive t-score indicates the presence of the peak and a negative t-score its absence. The best top-ranked peaks are in the 5–8 kDa bandwidth, and the 4 top-ranked are the most discriminant. Subtree A is indeed characterized by the presence of three of them (5228.8, 5917.8, and 8974.3 m/z) and the absence of peak 4996.9 m/z. Automatic checking of these 4 peaks against UniProtKB retrieved all of them, showing that peak 5228.8 is possibly an OxaA protein and that the absent peak may be a transposase.

## Discussion

The objective was to present a pipeline using MALDI-TOF spectra in the early stages of the situation diagnosis in order to disclose a temporo-proteic cluster that could suggest the existence of an epidemic chain as suggested by Sintchenko [25]. A previous study on *Staphylococcus saprophyticus* [14], allowed us to explore the capability of MALDI-TOF MS spectral clustering in epidemiology with the identification of a particular subspecies circulating in Marseille. From this attempt, we have progressively improved the stability and power of spectra analyses with a better control of the intra and inter automaton variations (additional alignment on the BTS peaks), less analysis noise (exclusion of core peaks), the adding of visualization cues by graphical representations contrasting homogeneous temporo-proteic clusters, and the identification and characterization of discriminant peaks. All these processes are possible because this pipeline is directly connected to a single system MIDaS that systematically collects and concentrates all the data from the microbiology laboratory, both the biological results associated with patient and sample information and the MALDI-TOF spectra. Nevertheless, this pipeline is usable only if the spectra have been performed under the same standardized conditions, whatever the culture medium used. This is an essential condition for comparing spectra within the same clustering process.

The carriage of a bacterial species in a human population is made of the cohabitation of a multitude of lineages corresponding to multiple chains of transmission. Each of them may have its own epidemiological characteristics [28–30]. This explains why genetic fingerprinting techniques such as whole-genome sequencing (WGS) are increasingly used in many epidemiological contexts, in particular for confirming that samples belong to a same epidemic chain [31] or for studying the dynamics of epidemics [8, 32–34]. We cannot ignore the fact that a same genome may have different phenotypic expressions, and conversely [35]. However, in the context of our study, we hypothesize that the phenotypic expression of a strain is a proxy for its genetic profile, insofar as its culture conditions are standardized (i.e. the environmental pressure being the same during bacterial growth). By extension, we consider that a set of bacterial strains presenting a same phenotypic expression can be sufficiently similar for belonging to a sample of the same epidemic process, and, with respect to the limitations presented above, a possible epidemic clone. The aim of this pipeline is not to identify a possible species subgroup, as it may be done using genomic subtyping, but to propose a low cost proxy to genomic typing with a same phenotypic subpopulation profile suggesting the existence of a epidemic transmission process (the spectra belonging to a sampling of this process) during the first steps of the epidemiological investigation. Therefore, the use of genetic methods is not essential and necessary at this moment. Nevertheless, to fully confirm these results, genetic or molecular methods remain needed and would be done during the continuation of the investigation.

The use of this new-generation pipeline in the case of an alarm issued for *Streptococcus pneumoniae* has made it possible to reveal a cluster of spectra with close proteinic and temporal distances. This subtree was characterized by the presence of three discriminant peaks (5228.8, 5917.8, and 8974.3 m/z) and the absence of peak 4996.9 m/z. A further investigation on UniProt KB showed that peak 5228.8 is possibly an OxaA protein and that the absent peak may be a transposase, additional hypothetical information that could indicate protein biomarkers of the virulence of the bacteria subpopulation and therefore a possible epidemic potential. The information we have at our disposal does not allow us to provide evidence of transmission between the patients. Transmission should be studied at a later stage during the next steps of the epidemiological investigation. No genetic sequencing was performed during this study to confirm the genetic affiliation of these spectra to the same epidemic subpopulation. Indeed, subtree A contains a majority of samples not kept by the laboratory due to the type of sample (sputum, skin swabs), making it impossible to sequencing a posteriori of the entire subtree A. Only two deep samples could be subjected to whole genome sequencing, which would make the interpretations questionable, reinforcing the need for a prospective analysis route.

The phenotypic expression in MALDI-TOF spectra may include an antibiotic resistance or a virulence factor. A more recent version of our system includes the possibility of a double clustering of the samples, taking in account several characteristics associated to the samples in the surveillance database, including the antibiotic resistance testing (Additional file 1: Fig. S1, Table S1). However, a resistance is usually associated to a bacterial or plasmid genomic feature, which is not systematically expressed by the production of a protein in the MALDI-TOF bandwidth. The usual position is to consider an indirect association, that is a specific clustering of the spectra is a cue of the belonging to a bacterial population subgroup, which is also characterized by a chemo resistance.

## Conclusions

This example shows how an adequate processing of the bacteria phenotypic expression by using the protein expression coming routinely at low cost by MALDI-TOF mass spectrometry [4, 10, 11] may show a spectra clustering that support a quasi-real time identification and characterization of clusters suggesting and providing essential information on a potentially epidemic situation. It is a valuable tool for epidemiological sensemaking and for deciding on the continuation of the epidemiological investigation, in particular the involving of additional costly resources to confirm or invalidate the alarm. Further studies are also in progress to evaluate at a large scale our approach on other community and nosocomial bacterial species during epidemiological surveillance. In the future, we can expect the spectra clustering will support a supplementary alarm detection algorithm. Some additional studies remain to be carried out to tackle this question.

## Supplementary Information

**Additional file 1: Fig. S1.** Clustering of phenotypic characters (top) with corresponding labels onthe abscissa associated with protein clustering on the right with correspondinglabels (sample number) on the ordinate. The red color indicates the presence ofthe phenotypic character. **DOX**: doxycycline; **E**: erythromycin; **CLI**:clindamycin; **1_4**: patients between 1 and 4 years old; **0**: newborn;**16_34**: patients between 16 and 34 years old; **FOS**: fosfomycin; **TEC**:teicoplanin; **PG**: penicillin G; **AMX**: amoxicillin; **ACM**: amoxicilin-ac.clavulanic;**CSF**: cerebro-spinal fluid; **5_15**: patients between 5 and 15 yearsold; **80_**: patients aged 80 years and over; **Death**: patients deathsduring hospitalization; **65_79**: patients between 65 and 79 years old; **Other**:other types of sample; **35_64**: patients between 35 and 64 years old; **Pulm**:pulmonary sample; **noso**: nosocomial sample; **mult**: infection byseveral agents; **ENT**: ear, nose, throat sample; **Comm**: communitysample. **Table S1.** Phenotypic characters of patients belongingto subtree A. 

## Data Availability

The data from our epidemiological surveillance are not available on the public domain, but anyone interested in using the data for scientific purpose is free to request permission from the corresponding author: Hervé Chaudet (herve.chaudet@gmail.com).

## Abbreviations

AP-HM: Assistance Publique – Hôpitaux de Marseille
BALYSES: Bacterial real-time Laboratory-based Surveillance System
BinDA: Binary discriminant analysis
BTS: Bacterial test standard
IHU-MI: Hospital University Institute Méditerranée infection
MALDI-TOF: Matrix assisted laser desorption ionization-time of flight
MIDaS: Mediterranée Infection Data Warehousing and Surveillance
MSP: Main spectrum profiles
REST: Representational state transfer
SNR: Signal to noise ratio
WGS: Whole-genome sequencing
