# Supplementary material for: Using MALDI-TOF spectra in epidemiological surveillance for the detection of bacterial subgroups with a possible epidemic potential
Source: BMC Infect Dis. 2021 Oct 28;21:1109. doi: 10.1186/s12879-021-06803-3 (PMC8554970; doi:10.1186/s12879-021-06803-3)
Supplement: Supplementary file 1 — Additional file 1: Fig. S1. Clustering of phenotypic characters (top) with corresponding labels onthe abscissa associated with protein clustering on the right with correspondinglabels (sample number) on the ordinate. The red color indicates the presence ofthe phenotypic character. DOX: doxycycline; E: erythromycin; CLI:clindamycin; 1_4: patients between 1 and 4 years old; 0: newborn;16_34: patients between 16 and 34 years old; FOS: fosfomycin; TEC:teicoplanin; PG: penicillin G; AMX: amoxicillin; ACM: amoxicilin-ac.clavulanic;CSF: cerebro-spinal fluid; 5_15: patients between 5 and 15 yearsold; 80_: patients aged 80 years and over; Death: patients deathsduring hospitalization; 65_79: patients between 65 and 79 years old; Other:other types of sample; 35_64: patients between 35 and 64 years old; Pulm:pulmonary sample; noso: nosocomial sample; mult: infection byseveral agents; ENT: ear, nose, throat sample; Comm: communitysample. Table S1. Phenotypic characters of patients belongingto subtree A. [file 12879_2021_6803_MOESM1_ESM.docx]

**Supplementary data**

**Supplementary figure S1**

**Figure S1** - Clustering of phenotypic characters (top) with corresponding labels on the abscissa associated with protein clustering on the right with corresponding labels (sample number) on the ordinate. The red color indicates the presence of the phenotypic character.

**DOX**: doxycycline; **E**: erythromycin; **CLI**: clindamycin; **1_4**: patients between 1 and 4 years old; **0**: newborn; **16_34**: patients between 16 and 34 years old; **FOS**: fosfomycin; **TEC**: teicoplanin; **PG**: penicillin G; **AMX**: amoxicillin; **ACM**: amoxicilin-ac.clavulanic; **CSF**: cerebro-spinal fluid; **5_15**: patients between 5 and 15 years old; **80_**: patients aged 80 years and over; **Death**: patients deaths during hospitalization; **65_79**: patients between 65 and 79 years old; **Other**: other types of sample; **35_64**: patients between 35 and 64 years old; **Pulm**: pulmonary sample; **noso**: nosocomial sample; **mult**: infection by several agents; **ENT**: ear, nose, throat sample; **Comm**: community sample.

**Table S1 – Phenotypic characters of patients belonging to subtree A**

| **Number of sample** | **ACM** | **CLI** | **CRE** | **DOX** | **E** | **FOS** | **GEN** | **ICM** | **PG** | **PT** | **RA** | **TEC** | **VAB** |
| --- | --- | --- | --- | --- | --- | --- | --- | --- | --- | --- | --- | --- | --- |
| Q201655277 | S | S | S | S | S | R | S | S | I | S | S | S | S |
| Q201657570 | NA | NA | NA | NA | NA | NA | NA | NA | NA | NA | NA | NA | NA |
| Q201661370 | S | S | S | S | S | S | S | S | S | S | S | S | S |
| Q201658830 | S | S | S | S | S | S | S | S | S | S | S | S | S |
| Q201659395 | S | R | S | R | R | R | S | S | I | S | S | S | S |
| Q201643799 | S | S | S | S | R | S | S | S | I | S | S | S | S |
| Q201648686 | S | - | S | S | S | S | S | S | S | S | S | S | S |

**ACM**: amoxicilin-ac.clavulanic

**CLI**: clindamycin

**CRE**: ceftriaxone E-test

**DOX**: doxycycline

**E**: erythromycin

**FOS**: fosfomycin

**GEN**: high dose gentamicin

**ICM**: imipenem E-test

**PG**: penicillin G

**PT**: pristinamycin

**RA**: rifampicin

**TEC**: teicoplanin

**VAB**: vancomycin E-test
